# Using Rheology to Understand Transient and Dynamic Gels

**DOI:** 10.3390/gels8020132

**Published:** 2022-02-18

**Authors:** Simona Bianco, Santanu Panja, Dave J. Adams

**Affiliations:** School of Chemistry, University of Glasgow, Glasgow G12 8QQ, UK; 2266630b@student.gla.ac.uk (S.B.); santanu.panja@glasgow.ac.uk (S.P.)

**Keywords:** gel, rheology, transient, dissipative, dynamic

## Abstract

Supramolecular gels can be designed such that pre-determined changes in state occur. For example, systems that go from a solution (sol) state to a gel state and then back to a sol state can be prepared using chemical processes to control the onset and duration of each change of state. Based on this, more complex systems such as gel-to-sol-to-gel and gel-to-gel-to-gel systems can be designed. Here, we show that we can provide additional insights into such systems by using rheological measurements at varying values of frequency or strain during the evolution of the systems. Since the different states are affected to different degrees by the frequency and/or strain applied, this allows us to better understand and follow the changes in state in such systems.

## 1. Introduction

In most cases, when gels are prepared, their properties ideally do not change with time. However, there is an increasing interest in gels which have controllably changing properties. Transient gels as well as gels that are formed by fueled systems are a growing field, partially driven by a generic interest in systems chemistry as well as by potential analogy with biological systems [1,2,3,4,5].

Several such systems have been reported that change phase with time [6,7,8,9,10,11,12,13,14,15,16,17,18]. There are examples where an initial solution (sol) state forms a gel in a pre-determined or programmable manner, then reverting to a sol state. Such sol-to-gel-to-sol systems can be driven by changes in pH, for example, by using enzymatic methods that result in well controlled and predictable rates of pH change [19,20,21,22]. Alternatively, such sol-to-gel-to-sol systems can be driven by the addition of a fuel that converts a non-gelator to a gelator; when the fuel runs out, the gelator reverts to a non-gelator and hence, the gel state only persists for as long as the fuel is available [3,23,24,25,26]. Other examples of transient or dynamic systems include gel-to-sol-to-gel systems [17,27] and gel-to-gel-to-gel [28] systems where changes in (for example) pH can result in the underlying network underpinning the gel changing.

Despite the number of examples of these transient and dynamic gels systems whereby changes in state occur, there is perhaps a surprising lack of rheological data showing the properties of these systems (although there are of course many examples where more traditional sol-to-gel transitions have been examined). In some cases, photographs of upturned vials are provided the only ‘proof’ of gel states [29,30].

Where rheological data are provided, these typically do not involve time-dependent measurements. For instance, these can be snapshots at some point during the process [24,31] where a frequency sweep has been measured whilst the system was evolving, or show that some examples are perhaps not true gels on the basis of the storage modulus (G′) and loss modulus (G″) measured [8], or for only G′ to be provided [26,27,32]. However, rheology should be a useful means of understanding the phase changes that occur during these sol-to-gel and gel-to-sol transitions over time.

We have previously found that using time sweep rheology can be an effective tool to monitor phase changes, but these are not always clear. For example, for a sol-to-gel-to-sol transition, we found that both G′ and G″ increased with time and then decreased again, with the overall trend agreeing with the visual observation as to when an invertible gel was formed [16]. However, during this entire process G′ remains greater than G″ with tan δ (defined as G″/G′) < 0.15 throughout the sol-to-gel-to-sol transition [16,17,33]. Hence, from time sweep rheology, this could be defined as a gel throughout the transitions. Similar observations have been made elsewhere for gels that change to a sol state on irradiation [34]. Here, we show that useful insights into such transitions can be found by carrying out time sweep rheology at different frequencies and strains. This not only allows to correlate visual observations of the systems, but also enables one to control final properties of the materials.

## 2. Results and Discussion

**Figure 1:**
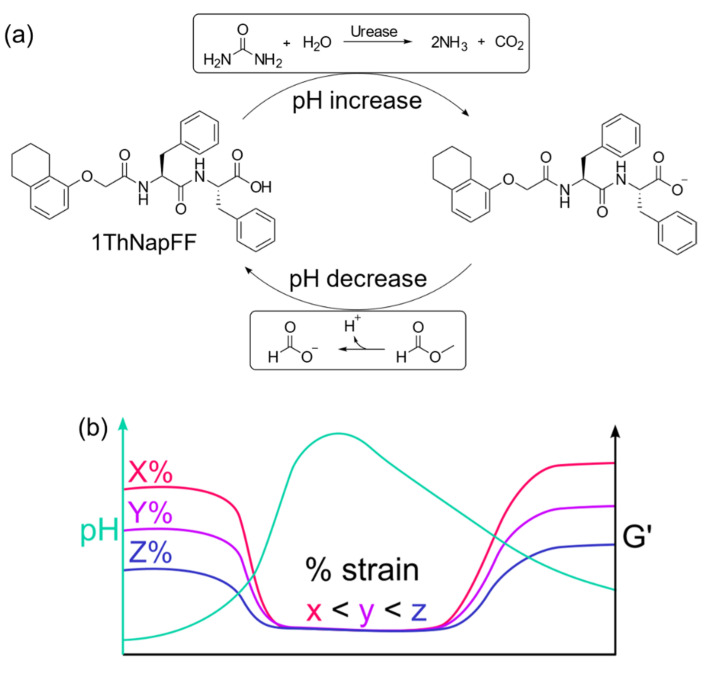
(**a**) Chemical structure of the gelator **1ThNapFF** used in the study. This compound undergoes deprotonation in presence of urea and urease due to production of ammonia. Base-catalysed hydrolysis of methyl formate reduces the pH and regenerates the structure of **1ThNapFF**. (**b**) Cartoon representing the effect of variation of strain on the G′ profile of a dynamic system undergoing gel-to-sol-to-gel transition. The final value of G′ decreases as the applied strain increases.

**Figure 2:**
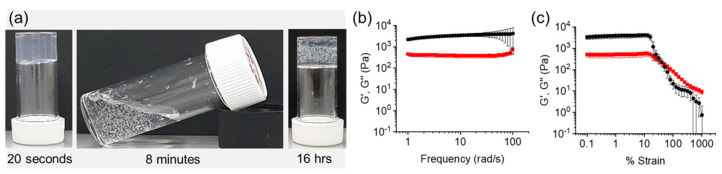
(**a**) Photograph of the gel-to-sol-to-gel transition brought about by the sequential increase and decrease in pH. Note that bubbles appear during the phase transitions which can be seen in the sol and final gel state; (**b**) Frequency sweep for a stable gel formed at low pH; (**c**) Strain sweep for a stable gel formed at low pH. For both (**b**,**c**), no enzyme, urea or methyl formate were added and hence, the solvent-triggered gel remains stable. In all cases, initial concentration of **1ThNapFF** = 2 mg/mL, urea = 0.01 M, urease = 0.2 mg/mL and volume of methyl formate is 100 μL. The black data represent G′ and the red data represent G″.

To exemplify the point, we chose a system we have described previously [17] whereby the gelator **1ThNapFF** (Figure 1a) is initially dissolved in DMSO at a concentration of 10 mg/mL. On addition of water such that the concentration of **1ThNapFF** is 2 mg/mL (DMSO–water solvent ratio is 20:80), a gel is formed due to the change in the properties of the solvent. Using this system, we can construct a gel-to-sol-to-gel process by incorporating urease (0.2 mg/mL), urea (0.01 M) and 100 µL of methyl formate. Within the first 20 min, the pH increases from the initial value of 4.43 to 7.52 driven by the conversion of urea to ammonia by urease [35]. Since **1ThNapFF** forms wormlike micelles at high pH, a sol phase transition from the initially formed gel at low pH occurs [17]. However, the hydrolysis of methyl formate becomes dominant at high pH, leading to a gradual pH decrease. Therefore, a pH-triggered gel is then formed once the pH falls below the apparent p*K*_a_ of the gelator (~6.3). After regelation, the system reaches a final pH of 5.62. Hence, overall we observe gel-to-sol-to-gel transitions (Figure 1 and Figure 2a shows photographs at different time points exemplifying this process).

To monitor the gelation with time, rheological data are collected at a set frequency and strain. Typically, the storage and loss moduli should be frequency independent for a gel. However, in the sol phase there will likely be a strong frequency dependence. Hence, collection data for the same system at different values of frequency at a set strain would be expected to result in differences in the sol phase especially. The strain used would typically be low, since the gels formed by such low molecular weight systems break at low strain; thus, to ensure data are within the linear viscoelastic region (LVE), one would typically use a value of <1%. Rheological data for stable gels at low pH (i.e., formed using a solvent-switch only, with no urease, urea or methyl formate added to change the pH) show that the LVE region for the initial gel extends to 12.5% (Figure 2b,c).

**Figure 3:**
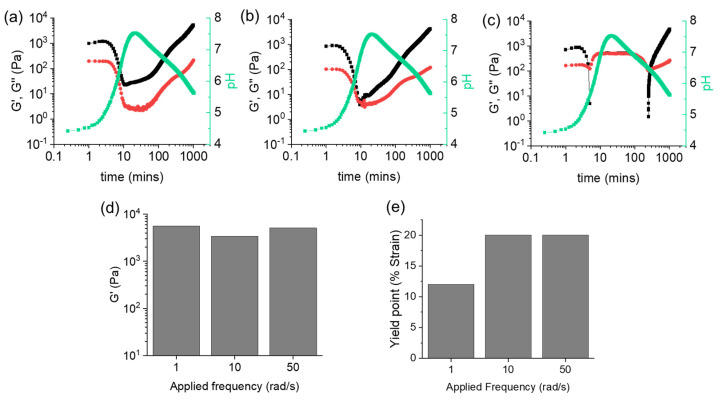
Variation of G′ (black), G″ (red), and pH (green) with time for **1ThNapFF** in presence of urea–urease reaction and methyl formate at (**a**) 1 rad/s, (**b**) 10 rad/s and (**c**) 50 rad/s. Throughout all measurements, the strain value was fixed at 0.5%. Comparisons of (**d**) final G′ and (**e**) yield points of the three systems. For (**d**), the values of G′ at 0.5% strain from the strain sweeps are considered. In all cases, initial concentration of **1ThNapFF** = 2 mg/mL, urea = 0.01 M, urease = 0.2 mg/mL and volume of methyl formate is 100 μL. Note that the pH and rheology measurements were performed with two different vials under identical conditions. The data were then compared to investigate the variation of rheological properties to that of the changes of pH with time.

The time sweeps were collected at a value of 0.5% strain, while varying the frequency values, as shown in Figure 3 (the data are also shown with a linear *x*-axis scale in Figure S1, Supplementary Materials). Overlaid are the pH data. By inspection of the time sweeps, initially there is a clear gel phase with G′ > G″. As the pH reaches around 6, the p*K*_a_ of the terminal carboxylic acid is reached, resulting in sufficient deprotonation to lead formation of micellar structures. When these measurements are carried out at low frequency (1 rad/s, Figure 3a), a decrease in G′ and G″ is observed during this transition, but G′ dominates over G″ throughout and G′ never becomes less than 20 Pa. As the pH decreases once again, a two-stage increase in both G′ and G″ can be seen, which is not uncommon for these kinds of systems. Even though after 100 min G′ is greater than G″, the system is still evolving which is evident from the further increase of rheological moduli with time. Hence, we performed the time sweeps for a longer time to allow the system to reach an equilibrium state where the values of G′ and G″ tend to reach a plateau. This was generally observed after almost 12 h (Figure 3 and Figure S1).

At a frequency of 10 rad/s (Figure 3b), G′ becomes approximately equal to G″ for a short period of time during pH increase, before regelation occurs in a similar two-stage increase of both rheological moduli. At 50 rad/s, a very different profile is seen (Figure 3c), whereby G′ quickly becomes less than G″ and drops below 0 Pa for a significant time period. Once the pH starts to decrease, G′ dominates again over G″. However, in this case, regelation occurs in a single stage increase of the rheological moduli. By time sweep rheology, different behaviors of G′ and G″ were noticed after the pH increase. We assign the overall transition to a gel-to-sol-to-gel. If we do not include methyl formate in the system such that there is a pH increase, but no further decrease, a solution phase is formed as confirmed visually and by rheology (Figure S2a). When methyl formate is also included, after 20 min the pH is above 7 and we would expect a solution phase to be formed. However, frequency sweeps of the intermediate materials after 20 min (Figure S2b) show complex behavior with a low frequency region where G′ is greater than G″ (the data at higher frequency cannot be collected due to issues with the samples being below the minimum elastic torque for this measurement system). This could imply that a true solution phase is not formed and perhaps here a gel-to-gel-to-gel assignment would be more appropriate. However, we highlight that the system is evolving over the course of this experiment and an immediate repeat measurement shows different data (Figure S2c); this evolving over the course of the frequency sweep makes the assignment difficult.

Hence, it follows that we are able to pull out more clearly the phase behavior by carrying out the time sweeps at different frequencies. For instance, more liquid-like response can be seen at higher frequencies. These results resemble the findings by Setz et al. who showed that for materials with liquid-like properties, increasing frequencies results in disappearance of the elastic component (G′) [36]. In all cases, a gel was obtained at the end, further confirmed from the frequency independence of G′ for all materials (Figure S3). Interestingly, the final values of G′ and G″ were considerably higher than for the initially formed gels. We previously suggested that the increase of stiffness in the final gel is due to annealing, which results in conversion of the initially formed kinetically trapped gel to a more homogeneous gel by establishing an optimal balance of the physical interactions between the molecules [17]. Further comparison of the final G′ (Figure 3d) shows very similar values, as would be expected considering we are in a gel phase at the end where these moduli should be frequency independent. However, collecting a strain sweep at the end of the time measurement shows some differences between the data collected on the gels, with the yield point (considered as the strain value at which the gel starts to break and the G′ starts to decrease) being affected by the frequency at which the time sweep was carried out (Figure 3e and Figure S4 in Supplementary Materials). Indeed, it appears that the gel collapses at a higher strain when higher frequency is applied during the measurement. This needs to be considered when reporting data and therefore, simply collecting data on the final gel after a time sweep may not be representative of the quiescent system.

**Figure 4:**
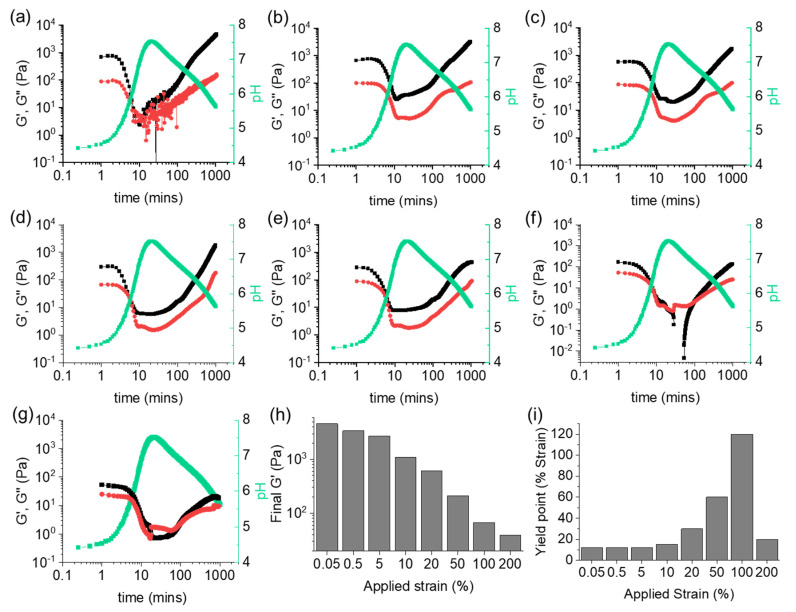
Variation of G′ (black), G″ (red), and pH (green) with time for **1ThNapFF** in the presence of urea–urease reaction and methyl formate at strain values of (**a**) 0.05%, (**b**) 5%, (**c**) 10%, (**d**) 20%, (**e**) 50%, (**f**) 100% and (**g**) 200%. Throughout all measurements, the frequency value was fixed at 10 rad/s. Comparisons of (**h**) final G′ and (**i**) yield point of the systems. For (**h**), the values of G′ at 0.5% strain from the strain sweeps are considered. In all cases, initial concentration of **1ThNapFF** = 2 mg/mL, urea = 0.01 M, urease = 0.2 mg/mL and volume of methyl formate is 100 μL. Note that the pH and rheology measurements were performed with two different vials under identical conditions. The data were then compared to investigate the variation of rheological properties to that of the changes of pH with time.

As the gels are not strain independent outside the LVE region, we would expect to observe a change in gelation behavior when the data are collected at a set frequency with different strain values (schematically shown in Figure 1b). The time sweeps performed at a constant frequency of 10 rad/s and varying strain are shown in Figure 4 and Figure S5. At low strain values of 0.05% and 0.5% (Figure 3b and Figure 4a), G′ and G″ are observed to decrease and briefly become equal as the pH increases. Then, regelation occurs in a two-stage process again when the pH decreases. Note here that due to the low response at 0.05% strain, low signal to noise ratio leads to the presence of noisy data around 10 to 100 min.

At a value of 5% strain (Figure 4b), we should observe little difference in behavior as the value is still within the LVE (Figure 2c). As the pH increases, G′ and G″ both decrease, though G′ and G″ never cross prior to regelation. The final G′ is lower than that observed when the data were collected at 0.05% and 0.5% strain, indicating that higher perturbation of the system affects the final properties. Therefore, we investigated the effect of applying higher strain to the system. At 10% strain (Figure 4c), a similar profile to 5% strain is seen with no crossover in G′ and G″. This is to be expected since this value is still below the yield point. Nevertheless, slight differences in the regelation profile are observed. In particular, the final G′ value was around 50% less compared to the value when the data were collected at a strain of 5%.

Different profiles were observed when we applied strain during the time sweep at values outside of the LVE region of the initial gel (Figure 2c). When the strain was increased to 20%, the initial G′ appeared at a lower value than for the low strain data due to increased perturbation of the gel network (Figure 4d). Both G′ and G″ decrease during pH uptake and overlap for a short period of time. However, no crossover point between the rheological moduli was observed and G′ > G″ throughout the rest of the measurement. Compared to the other samples, the two-stage increase in G′ and G″ was not observed upon regelation, with a more linear uptake of the values. A similar profile is observed for 50% strain with a significantly lower value for the final G′. This suggests that a weaker gel is formed under such conditions (Figure 4e). At 100% strain, G′ briefly drops below 0 Pa after 30 min (Figure 4f). As the pH decreases, regelation is observed, though the final G′ and G″ occur at lower values than the initial ones, differently from all other measurements. To further test the strain limit of this system, a time sweep was performed at 200% strain (Figure 4g). Although a similar profile to 100% strain was observed within the first 10 min, the final G′ and G″ were seen at very close values (tan δ = 0.53).

Taken together, we again note that the behaviors of G′ and G″ absolutely depend on the applied strain during a gel-to-sol-to-gel transition. Again, frequency sweeps carried out after 20 min were complicated and suggested in some cases gel-like behavior although in most cases G′ and G″ were very close (Figure S6). The final materials obtained after the time sweeps were established to be gels from the frequency sweep (G′ > G″), although a slight frequency dependency of the G′ for the gels prepared outside the LVE region was noticed (Figure S7). However, the final mechanical properties of the hydrogel are clearly influenced by applying different strain, with decreasing stiffness as the strain increases (Figure 4h). The applied strain is also found to affect the strain sweep behavior of the gels, where a trend in the yield point is observed (Figure 4i and Figure S8 in Supplementary Materials). Thus, this needs to be further taken into consideration when characterizing these dynamic systems.

## 3. Conclusions

Overall, we have shown here that systems that evolve between solution and gel states can be probed effectively using time sweep rheology. The data collected depend on the absolute frequency and strain at which the data are collected as gels and solutions have a different frequency dependence. Depending on the values at which the rheology is collected, it is therefore possible for the system to appear as if there is a gel phase present throughout (when the data are collected at low frequencies) with G′ > G″ at all times, even though visual observation shows that a solution phase is present. Collecting data at higher frequencies more effectively pulls out the phase transitions with a crossover between G′ and G″. Collecting data at different strains shows that there can be effects on the mechanical properties even when the measurement is carried out at a strain within the initial LVE. Here, the system evolves from a gel to a sol to a gel; as the gel starts to break apart, presumably the system’s response to strain is highly dependent on the time and hence phase present. Moreover, for such pH-driven dynamic systems, the final material properties are typically controlled by varying the rate of pH change [17]. Here for the first time, we showed that the properties of the final gels can also be controlled by varying the applied rheological conditions under a fixed rate of pH change. Self-regulating dynamic reconfiguration of gel properties often leads to materials that cannot be prepared directly [17,37]. In this endeavor, the insights provided in this work would not only be helpful to understand the self-regulating dynamic systems but also enable to prepare a wide variety of materials from a single starting gel using rheology.

## 4. Materials and Methods

**Materials.** The **1ThNapFF** gelator was synthesized as described previously [38]. Urease (U4002-100KU, Jack Beans, 100,000 units/g solid) and urea (ultrapure 99%) were purchased from Alfa Aesar. All other chemicals and solvents were obtained from commercial suppliers and used as received. Deionised water was employed in all the experiments.

**Preparation of solutions.** To prepare the stock solution, the gelator **1ThNapFF** was dissolved by stirring in DMSO at a concentration of 10 mg/mL. A stock solution of the enzyme was prepared to achieve a final concentration of 0.253 mg/mL. The concentration was calculated by taking the mass of the enzyme (in mg) in a known volume of H_2_O. Stock solution of urea at a concentration of 2 M was prepared in H_2_O. Both the urea and urease were highly soluble in water and thus did not require stirring.

To induce the gel-to-sol-to-gel transitions, the enzyme-catalyzed reactions with **1ThNapFF** were performed in presence of methyl formate. For these gels, 400 μL of the gelator solution, 10 μL of urea and 100 μL of methyl formate were transferred into a 7 mL Sterilin vial. 1.580 mL of the urease solution was then added to the mixture in one aliquot and the system was left undisturbed. The solvent ratio of DMSO and H2O was kept at 20:80. Initial concentrations of the components were as follows: **1ThNapFF** = 2 mg/mL, urea = 0.01 M, urease = 0.2 mg/mL and volume of methyl formate is 100 µL.

**Hydrogel preparation. 1ThNapFF** hydrogels were firstly prepared in absence of enzyme and methyl formate. For this method, 1.6 mL of H_2_O were transferred to 0.4 mL of the gelator solution in DMSO (final concentration 2 mg/mL). The samples were left overnight prior to any measurements.

For the hydrogels prepared in presence of the enzyme and methyl formate, the procedure highlighted above was followed.

**Rheological measurements.** All rheological experiments were carried out on an Anton Paar Physica MCR 301 rheometer at a constant temperature of 25 °C. For all measurements, a vane and cup geometry (ST10-4V-8.8/97.5-SN42404) was employed at a measuring distance of 2.1 mm. Strain sweeps were performed over the range of 0.01% to 1000% strain at a frequency of 10 rad/s. Frequency sweeps were taken at 0.5% strain while ramping up the frequency from 1 rad/s to 100 rad/s. Time sweeps were performed overnight with variable parameters for strain and frequency. The samples were prepared immediately before positioning the vial in the rheometer cup system. In the first set of measurements, the strain was kept constant at 0.5% and the time sweep was performed at frequency values of 1 rad/s, 10 rad/s and 50 rad/s. Time sweeps were then carried out while keeping the frequency constant at 10 rad/s and at strain values of 0.05%, 0.5%, 5%, 10%, 20%, 50%, 100%, 200%. Strain and frequency sweeps for the gels obtained involving gel-sol-gel transitions were conducted just after the time sweeps without lifting the vane from the system. The angular frequency was kept at 10 rad/s for all strain sweeps and the strain was kept at 0.5% for all frequency sweeps.

To confirm the nature of the materials obtained after the initial decrease of G′ and G″ during time sweeps, we performed frequency sweep experiments on the samples after 20 min of running the time sweeps without lifting the vane from the system. Throughout all frequency sweeps, the strain value was 0.5%.

**pH measurements.** pH measurements were performed using a HANNA FC200 pH probe with a 6 mm × 10 mm conical tip. The accuracy of the pH values was ±0.1. To monitor the pH change of the system with urea-urease and methyl formate, the reaction mixture was prepared as above immediately before measurement. The temperature was kept at 25 °C by using a circulating water bath.

## Data Availability

All the necessary data can be found in the Supplementary Materials.

## Supplementary Materials

The following supporting information can be downloaded at: https://www.mdpi.com/article/10.3390/gels8020132/s1, Figures S1–S9: tan δ data and further time, frequency and strain sweeps.
