# Supplementary material for: Using Rheology to Understand Transient and Dynamic Gels
Source: Gels. 2022 Feb 18;8(2):132. doi: 10.3390/gels8020132 (PMC8872063; doi:10.3390/gels8020132)
Supplement: Supplementary file 1 [file gels-08-00132-s001.zip › gels-1604228-supplementary.pdf]

gels

MDPI

Supplementary Materials

Using rheology to Understand Transient and Dynamic Gels

Simona Bianco, Santanu Panja and Dave J. Adams *

School of Chemistry, University of Glasgow, Glasgow G12 8QQ, UK; 2266630b@student.gla.ac.uk (S.B.); santanu.panja@glasgow.ac.uk (S.P.)

* Correspondence: dave.adams@glasgow.ac.uk

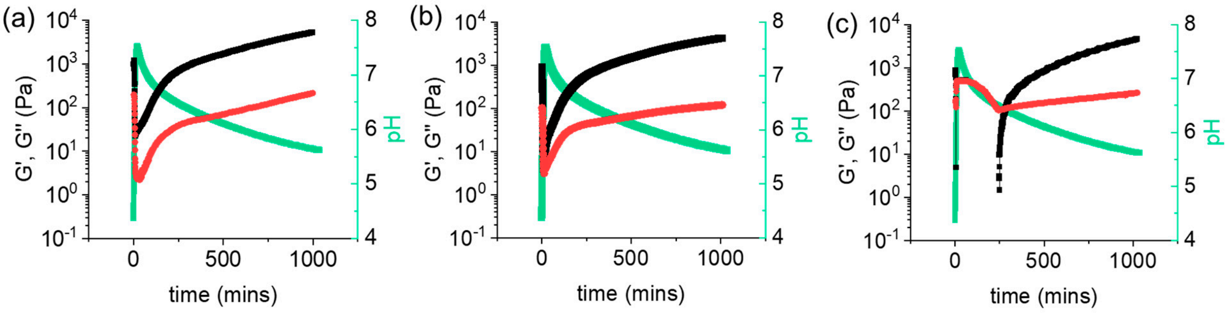

Figure S1. Variation of G′ (black), G″ (red), and pH (green) with time for 1ThNapFF in presence of urea- urease reaction and methyl formate at (**a**) 1 rad/s, (**b**) 10 rad/s and (**c**) 50 rad/s. Throughout all measurements, the strain value was fixed at 0.5%. In all cases, initial concentration of [1ThNapFF] = 2 mg/mL, [urea] = 0.01 M, [urease] = 0.2 mg/mL and volume of methyl formate is 100 μL. Figure S1 represents Figure 3 in the manuscript in linear x-scale.

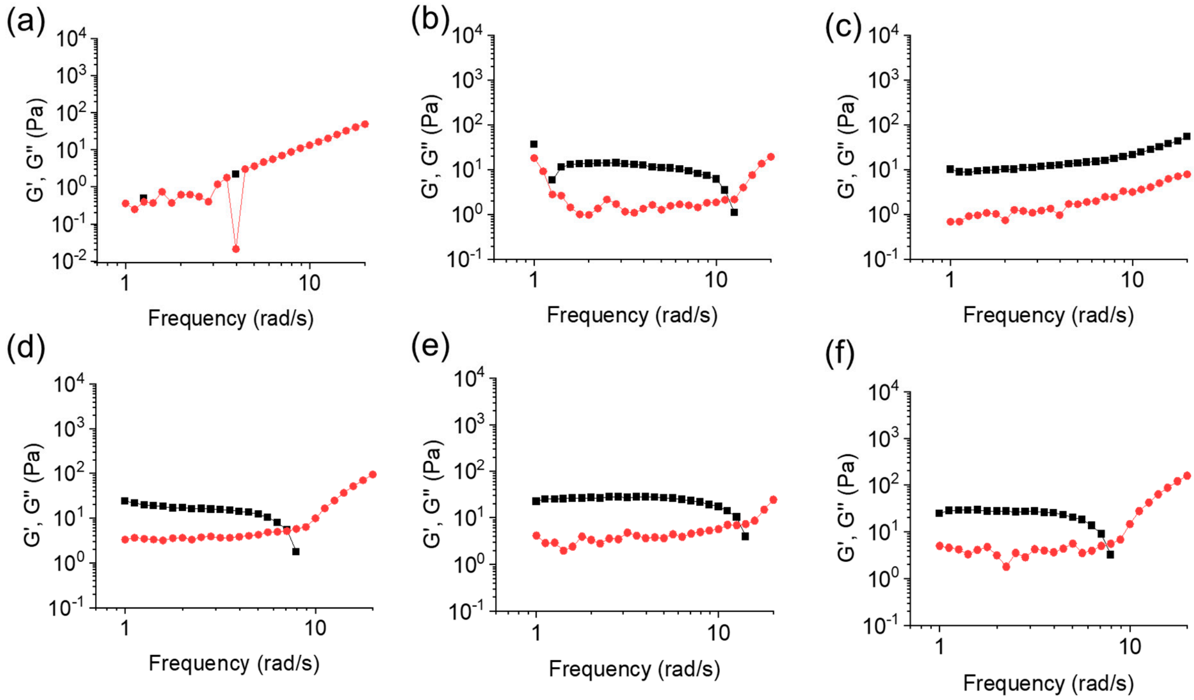

Figure S2. (**a**) Frequency sweep of the **1ThNapFF** system with no methyl formate after 45 minutes where the pH is 8.98. Visually, this system behaves as a liquid, and this is confirmed by the frequency sweep. (**b**) Frequency sweep of the **1ThNapFF** system obtained involving the enzymatic reaction in presence of methyl formate after initial gel-to-sol transition. The measurements were performed after running time sweeps for 20 mins at 0.5% strain at 10 rad/s. (**c**) Frequency sweep

collected immediately after the data collection for (**b**) showing that the system has evolved over the timescale of the first frequency sweep. (**d**) Frequency sweep carried out after the system has evolved for 20 minutes at 0.5% strain and angular frequency of 1 rad/s; (**e**) Frequency sweep carried out after the system has evolved for 20 minutes at 0.5% strain and angular frequency of 10 rad/s; (**f**) Frequency sweep carried out after the system has evolved for 20 minutes at 0.5% strain and angular frequency of 50 rad/s. In all cases, initial concentration of [**1ThNapFF**] = 2 mg/mL, [urea] = 0.01 M, [urease] = 0.2 mg/mL. For (**b**)-(**f**), the volume of methyl formate is 100 μL. The black symbols represent G′, the red symbols G″.

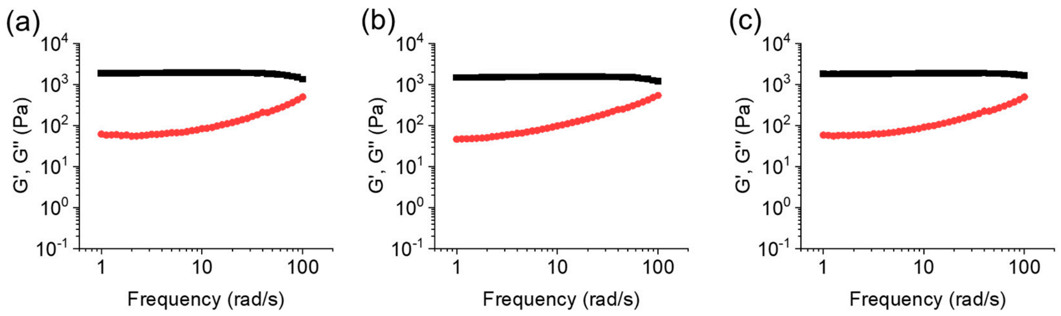

Figure S3. Frequency sweeps of the **1ThNapFF** system obtained involving the enzymatic reaction in presence of methyl formate. Table 0. strain and angular frequency of (**a**) 1 rad/s, (**b**) 10 rad/s and (**c**) 50 rad/s. In all cases, initial concentration of [**1ThNapFF**] = 2 mg/mL, [urea] = 0.01 M, [urease] = 0.2 mg/mL and volume of methyl formate is 100 μL. The black symbols represent G′, the red symbols G″.

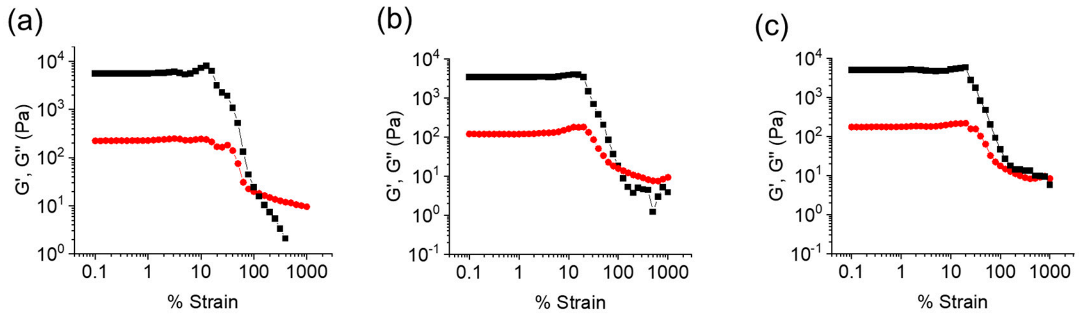

Figure S4. Strain sweeps of the hydrogels of **1ThNapFF** obtained involving the enzymatic reaction in presence of methyl formate. The gels are obtained after the time sweeps performed at 0.5% strain and angular frequency of (**a**) 1 rad/s, (**b**) 10 rad/s and (**c**) 50 rad/s. In all cases, initial concentration of [**1ThNapFF**] = 2 mg/mL, [urea] = 0.01 M, [urease] = 0.2 mg/mL and volume of methyl formate is 100 μL. The black symbols represent G′, the red symbols G″.

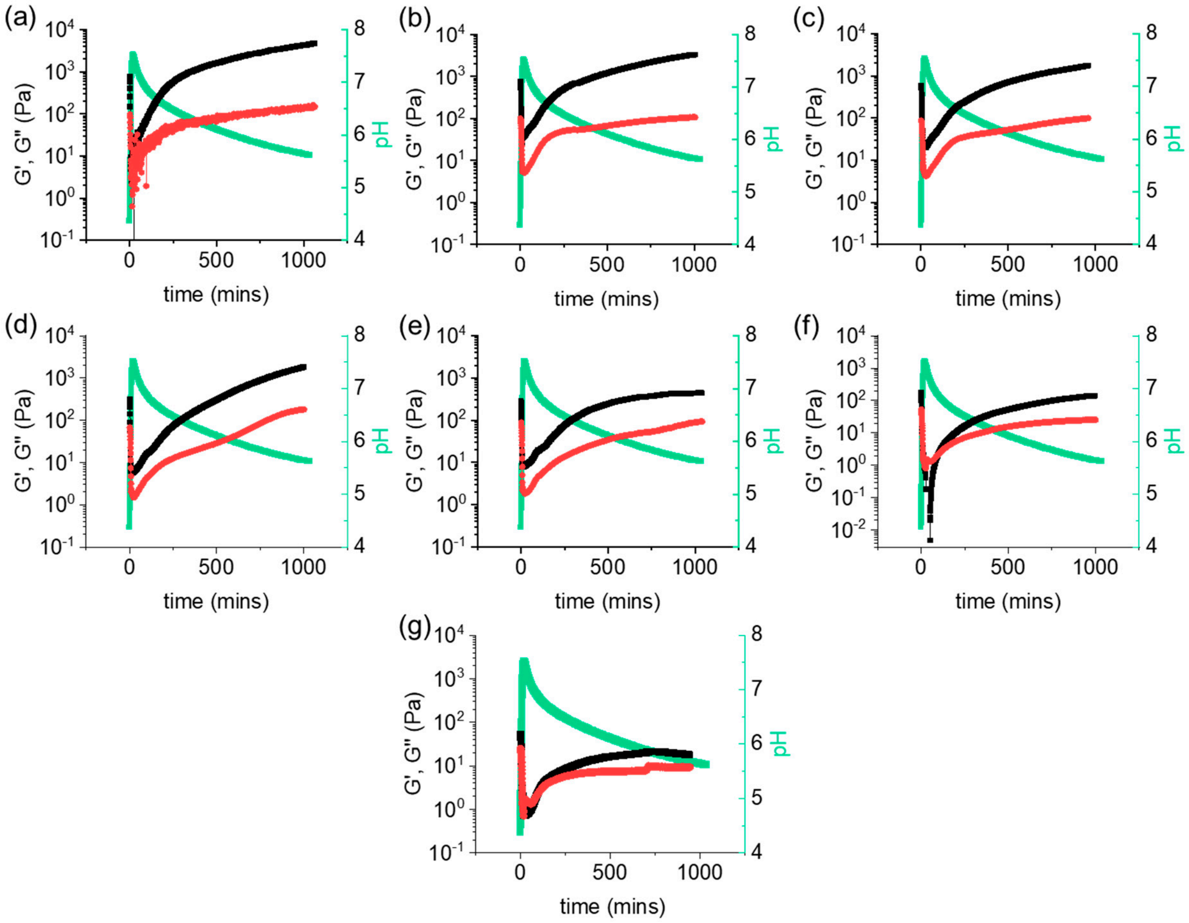

Figure S5. Variation of G′ (black), G″ (red), and pH (green) with time for 1ThNapFF in presence of urea- urease reaction and methyl formate at strain values of (**a**) 0.05%, (**b**) 5%, (**c**) 10%, (**d**) 20%, (**e**) 50%, (**f**) 100% and (**g**) 200%. Throughout all measurements, the frequency value was fixed at 10 rad/s. In all cases, initial concentration of [1ThNapFF] = 2 mg/mL, [urea] = 0.01 M, [urease] = 0.2 mg/mL and volume of methyl formate is 100 μL. Figure S5 represents Figure 4 in the manuscript in linear x-scale.

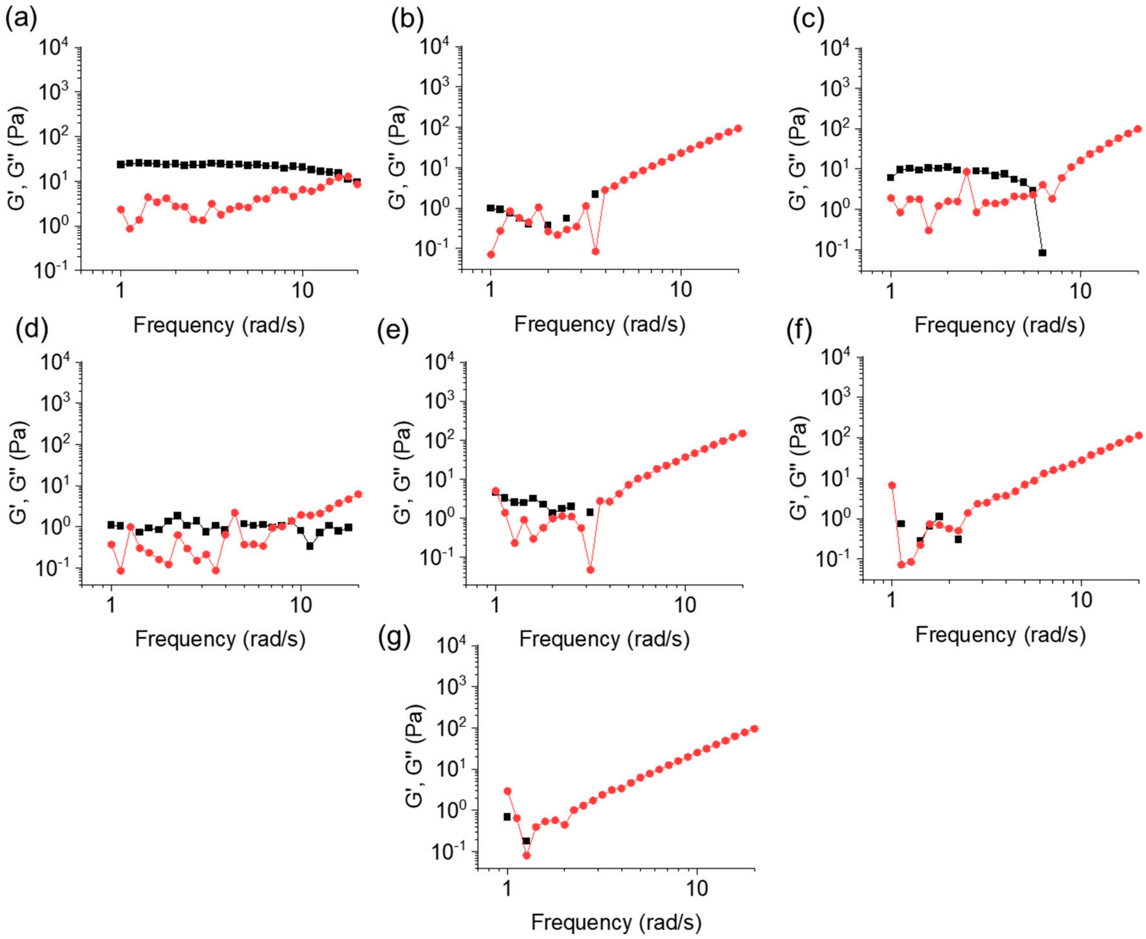

Figure S6. Frequency sweeps of the **1ThNapFF** system obtained involving the enzymatic reaction in presence of methyl formate after initial gel-to-sol transition. The measurements were performed after running time sweeps for 20 mins at an angular frequency of 10 rad/s and a strain of (**a**) 0.05%, (**b**) 5%, (**c**) 10%, (**d**) 20%, (**e**) 50%, (**f**) 100% and (**g**) 200%. In all cases, initial concentration of [**1ThNapFF**] = 2 mg/mL, [urea] = 0.01 M, [urease] = 0.2 mg/mL and volume of methyl formate is 100 μL. The black symbols represent G′, the red symbols G″.

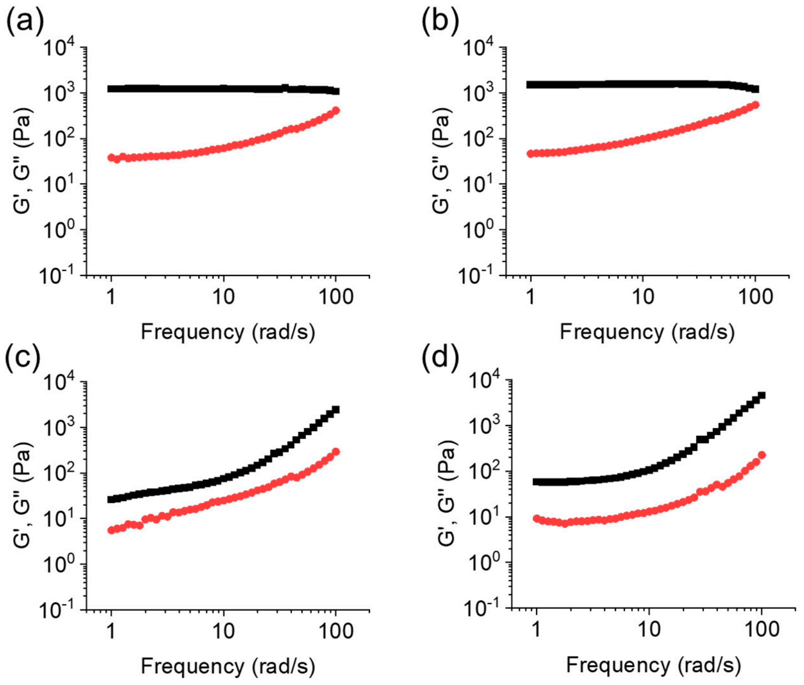

Figure S7. Frequency sweeps of the **1ThNapFF** system obtained involving the enzymatic reaction in presence of methyl formate. The gels are obtained after the time sweeps performed at an angular frequency of 10 rad/s and a strain of (**a**) 0.05%, (**b**) 0.5%, (**c**) 100%, (d) 200%. In all cases, initial concentration of [**1ThNapFF**] = 2 mg/mL, [urea] = 0.01 M, [urease] = 0.2 mg/mL and volume of methyl formate is 100 μL. The black symbols represent G′, the red symbols G″.

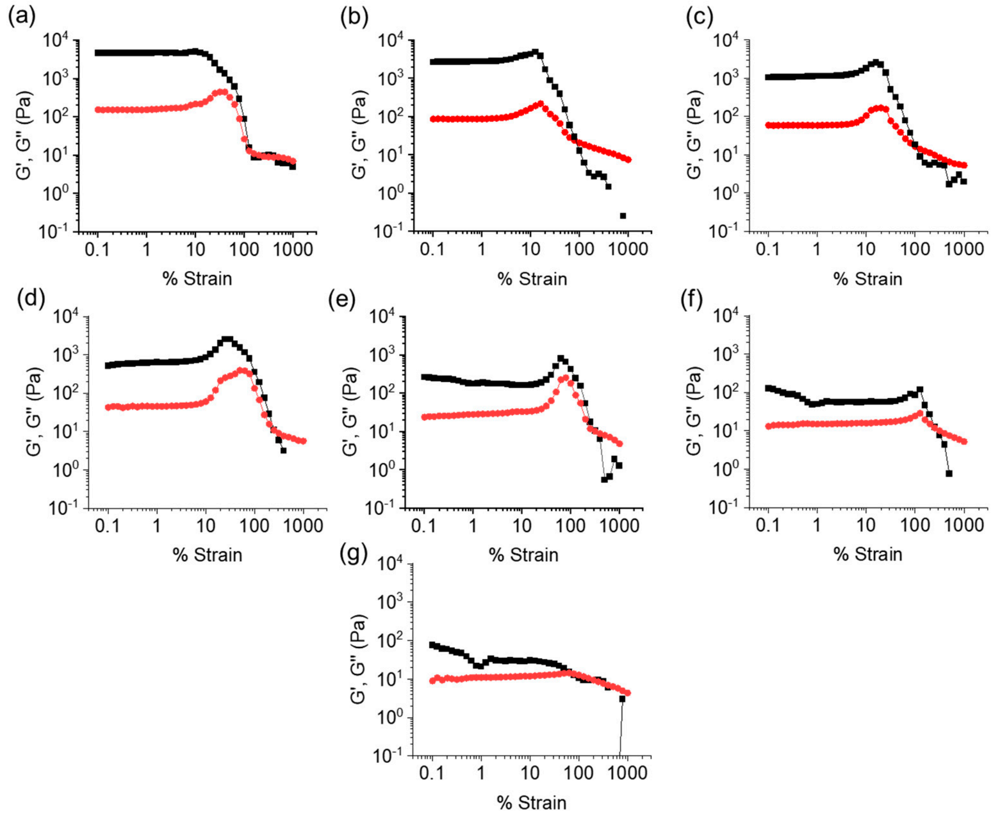

Figure S8. Strain sweeps of the hydrogels of **1ThNapFF** obtained involving the enzymatic reaction in presence of methyl formate. The gels are obtained after the time sweeps performed at an angular frequency of 10 rad/s and a strain of (**a**) 0.05%, (**b**) 5%, (**c**) 10%, (**d**) 20%, (**e**) 50%, (**f**) 100% and (**g**) 200%. In all cases, initial concentration of [**1ThNapFF**] = 2 mg/mL, [urea] = 0.01 M, [urease] = 0.2

mg/mL and volume of methyl formate is 100 μL. The black symbols represent G′, the red symbols G″.

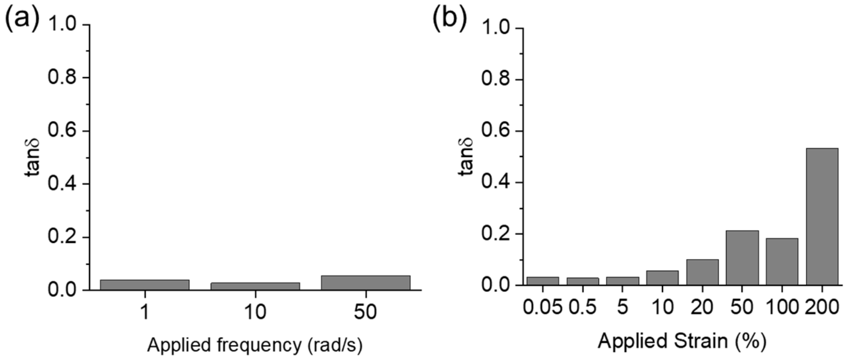

Figure S9. Bar graphs showing the final tanδ of **1ThNapFF** involving the enzymatic reaction in presence of methyl formate. The values were obtained from the last data point of the time sweeps performed at (**a**) 0.5% strain with varying frequency and (**b**) 10 rad/s frequency with varying strain values. In all cases, initial concentration of [**1ThNapFF**] = 2 mg/mL, [urea] = 0.01 M, [urease] = 0.2 mg/mL and volume of methyl formate is 100 μL.
